# The relationship between *elongation of very long-chain fatty acids (ELOVL) 2* polymorphism rs953413 and blood *n*-3 HUFA levels: a secondary analysis of EPA treatment in the seAFOod polyp prevention trial

**DOI:** 10.1186/s12263-026-00797-w

**Published:** 2026-05-14

**Authors:** Ge Sun, John R. Davies, Tracey Mell, Mark Harland, Rasha N. M. Saleh, Amanda D. Race, Paul M. Loadman, Anne Marie Minihane, Mark A. Hull

**Affiliations:** 1 Leeds Institute of Medical Research, University of Leeds, Leeds, UK; 2 Nutrition and Preventive Medicine, Norwich Medical School, University of East Anglia, Norwich, UK; 3 Department of Clinical and Chemical Pathology, Faculty of Medicine, Alexandria University, Alexandria, Egypt; 4 Institute of Cancer Therapeutics, University of Bradford, Bradford, UK; 5 Norwich Institute of Health Ageing, Norwich, UK; 6 Division of Gastrointestinal and Surgical Sciences, Leeds Institute of Medical Research, St James’s University Hospital, University of Leeds, Leeds, LS9 7TF UK

**Keywords:** Eicosapentaenoic acid, Elongase, Omega-3 polyunsaturated fatty acids

## Abstract

**Background:**

Individuals receiving a standardised daily dose of 2000 mg of the *n*-3 highly unsaturated fatty acid (HUFA) eicosapentaenoic acid (EPA) for 12 months in the seAFOod polyp prevention trial demonstrated wide inter-individual variability in red blood cell (RBC) EPA levels (expressed as % of total measured fatty acids). Eicosapentaenoic acid can be converted to *n*-3 docosapentaenoic acid (*n*-3DPA) by the elongase *elongation of very long-chain fatty acids (ELOVL) 2*. We tested whether single nucleotide polymorphisms (SNPs) in *ELOVL2* (rs3734398, rs2236212, rs953413, rs3798713, rs9393903) are associated with differential RBC EPA and *n*-3DPA levels in seAFOod trial participants at baseline and while receiving EPA supplementation.

**Results:**

Six hundred and three seAFOod trial participants had *ELOVL2* genotype and RBC *n*-3 HUFA data. All five *ELOVL2* SNPs were in strong linkage disequilibrium (R^2^> 0.95). Therefore, we focussed on rs953413 (genotype frequency: GG 30.8%, GA 50.1% and AA 19.1%), as the best characterised SNP, which is believed to be functional in human liver cells. Prior to trial intervention, minor (A) homozygotes displayed higher levels of *n*-3DPA (*P* < 0.01), but not EPA (*P* > 0.05), in RBCs than major (G) allele carriers. The trend towards a greater increase in *n*-3DPA values in AA homozygotes during EPA treatment compared with GA heterozygotes or GG homozygotes did not reach statistical significance. There was also no significant difference in the change in RBC EPA or DHA levels during EPA treatment according to rs953413 genotype.

**Conclusions:**

A secondary analysis of the seAFOod trial found that minor (A allele) homozygotes for the *ELOVL2* SNP rs953413 have a higher baseline RBC *n*-3DPA level than G allele carriers. However, rs953413 genotype did not significantly influence ΔEPA, Δ*n*-3DPA, or ΔDHA levels during 6 or 12 months of high-dose EPA supplementation. Therefore, we do not provide evidence that genetic variation in *ELOVL2* contributes to the inter-individual variability in the circulating EPA level associated with high-dose EPA supplementation.

## Introduction

Genetic variation can influence the metabolic response to nutritional interventions, including supplementation with long-chain *n*-3 highly unsaturated fatty acids (HUFAs) such as C20:5*n*-3 eicosapentaenoic acid and C22:6*n*-3 docosahexaenoic acid (DHA). In the placebo-controlled 2 × 2 factorial seAFOod polyp prevention trial, there was wide inter-individual variability in red blood cell (RBC) % EPA levels (expressed as % total measured fatty acids) during EPA treatment with a standardised daily dose of 2000 mg 99% pure EPA and/or aspirin 300 mg for 12 months [1]. While differences in baseline RBC EPA level, capsule compliance, and EPA formulation contributed to the variable response to EPA supplementation [2], metabolism of EPA to other *n*-3 HUFAs could contribute to the differential response to EPA supplementation. Levels of C22:5*n*-3 docosapentaenoic acid (*n*-3DPA), which is the intermediate product before desaturation to DHA during conversion of EPA to DHA, increased in RBCs from seAFOod trial participants randomised to EPA capsules in a bimodal manner [2]. However, there was no change in DHA levels during treatment [2]. This suggests that conversion of EPA to *n*-3DPA by the elongase enzyme *elongation of very long-chain fatty acids (ELOVL) 2* may be the key metabolic step contributing to variable RBC EPA levels in individuals receiving an identical dose of EPA. This hypothesis is supported by several studies that have reported the association between single nucleotide polymorphisms (SNPs) in the *ELOVL2* gene (including rs953413, rs2236212 and rs3734398) and low circulating DHA levels [3].

We have previously reported genotype-treatment interactions for polymorphisms in *fatty acid desaturase 2* (*FADS2*) [4] and oxylipin-related genes such as *Prostaglandin *G/H* synthase* [*PTGS*, also known as *cyclooxygenase*]1 and *PTGS2*) in the seAFOod trial [5]. In the latter study, we characterised 78 SNPs in multiple genes including SNP rs953413 (a functional SNP in a liver-specific enhancer element in the first intron of *ELOVL2*, which is the *ELOVL2* polymorphism that has been studied most extensively) [3, 6]. Therefore, we tested the hypothesis that *ELOVL2* SNP genotypes are associated with the change in RBC EPA level in seAFOod trial participants receiving EPA supplementation.

## Methods

### Study approval and registration

This work was performed as part of the STOP-ADENOMA study, which obtained approval from the London and Surrey Borders Research Ethics Committee (19/LO/1655) and is registered as ISRCTN05926847 (registration date 6th May 2011) as a sub-study of the seAFOod polyp prevention trial (protocol added 10th May 2022).

### Existing seAFOod trial data

Blood sampling during the seAFOod trial has been described in detail [7]. Blood sampling was scheduled at trial entry before Investigational Medicinal Product (EPA or placebo capsules and/or aspirin or placebo tablet) was started (visit [V] 1), after 6 months treatment (V4) and at the end of the 12-month intervention period just before colonoscopy (V6).

A panel of 9 fatty acids, including EPA and *n*-3DPA, were quantified using liquid chromatography-tandem mass spectrometry (LC-MS/MS) [8]. Relative *n*-3 HUFA levels in RBCs are expressed as the percentage of total measured fatty acids.

### Single nucleotide polymorphisms in ELOVL2

A secondary genetic analysis of SNPs in genes controlling oxylipin synthesis and degradation in 647 seAFOod trial participants has already been published [5]. The microfluidic multiplex SNP analysis also included five SNPs in *ELOVL2* (rs3734398, rs2236212, rs953413, rs3798713, rs9393903), which all satisfied Hardy-Weinberg equilibrium (HWE) in the original trial cohort used for genetic analysis [5].

### Statistical analysis

The percentage *n*-3 HUFA content of RBCs (EPA, DPA, and DHA) is presented as the median value and the interquartile range (IQR). Baseline RBC *n*-3 HUFA levels were investigated for the rs953413 genotypes (major homozygous GG, heterozygous GA, minor homozygous AA) for the whole seAFOod trial population before the trial intervention with EPA, with or without aspirin. Data distribution was analysed by the Shapiro-Wilk test and Q-Q plots, which confirmed that PUFA data were not normally distributed, although Levene’s test confirmed that PUFA data variance was equal across rs953413 genotypes. Therefore, RBC *n*-3 HUFA levels and the ELOVL2 product (*n*-3DPA)-to-precursor (EPA) ratio in AA homozygotes were compared with the other genotypes by the non-parametric univariate Kruskal-Wallis test given that other co-variates such as age and sex were balanced between the different genotype subgroups inside the randomised trial. For analysis of differences in the change in RBC *n*-3 HUFA level during EPA treatment for 6 and 12 months (Δ*n*-3 HUFA) according to rs953413 genotype, we restricted analysis to those participants that received EPA only, without concurrent aspirin treatment, in order to avoid any potential, indirect confounding effect of aspirin on *n*-3 HUFA metabolism. Individual Δ*n*-3 HUFA values for minor AA homozygotes were compared with the other rs953413 genotypes using the pairwise Wilcox test with Benjamini-Hochberg adjustment. Statistical significance was defined as *p* ≤ 0.05. All analysis was conducted using R Studio, version 2021.09.0.

## Results

Six hundred and three seAFOod trial participants had *ELOVL2* SNP data and RBC *n*-3 HUFA data available at baseline. All five *ELOVL2* SNPs satisfied HWE in the *n* = 603 cohort and were in strong linkage disequilibrium (particularly rs3734398, rs2236212, rs953413 and rs3798713 - all R^2^> 0.95). Therefore, we focussed on rs953413, as the best characterised SNP, which is believed to be functional in human liver cells, the primary site of long-chain *n*-3 HUFA synthesis and metabolism [6].

The rs953413 genotype frequency in 603 seAFOod trial participants with both *ELOVL2* genotype and RBC *n*-3HUFA data was GG 30.8%, GA 50.1% and AA 19.1% (Table 1). The distribution of rs953413 genotypes in the predominantly White European seAFOod trial cohort is consistent with that observed in Europeans in the 1000 Genomes Project (https://www.internationalgenome.org; accessed 9th October 2025).

**Table 1:** *n*-3 HUFA levels according to rs953413 genotype in seAFOod trial participants

	rs953413 genotype (n;%)	EPA (% total fatty acids)^1^	p^2^	ΔEPA^3,4^	p^5^	DPA (% total fatty acids)^1^	p^2^	ΔDPA^3,4^	p^5^	DHA (% total fatty acids)^1^	p^2^	ΔDHA^3,4^	p^5^
Baseline	GG (*n* = 186;30.8)	0.47(0.26,0.70)	0.21			1.08(0.57,1.66)	< 0.01			1.97(1.03,2.80)	0.71		
	GA (*n* = 302;50.1)	0.44(0.25,0.71)	0.07			1.15(0.61,1.69)	< 0.001			1.87(0.94,2.88)	0.24		
	AA (*n* = 115;19.1)	0.51(0.35,0.83)				1.51(0.89,2.11)				2.12(1.25,3.13)			
6 mo^6^	GG (*n* = 35;30.2)	1.78(0.90,2.76)	1.0	1.10(0.34,2.17)	0.89	2.27(1.09,3.12)	1.0	0.75 (0.21,2.02)	0.46	1.90(1.35,2.34)	0.7	-0.14(-0.85,0.25)	0.13
	GA (*n* = 58;50.0)	1.93(0.92,2.84)	1.0	1.43(0.54,2.32)	0.87	2.35(0.81,3.31)	1.0	1.0 (0.22,2.27)	0.46	1.57(0.59,2.49)	1.0	-0.17(-0.83,0.54)	0.09
	AA (*n* = 23;19.8)	1.73(1.20,2.45)		1.22(0.20,1.99)		1.95(1.2,3.1)		0.57 (-0.13,1.83)		1.39(0.73,1.96)		-0.65(-1.52,0.13)	
12 mo^6^	GG (*n* = 31;28.2)	1.38(0.82,2.18)	1.0	0.99(0.13,1.58)	0.62	2.29(1.68,3.47)	1.0	1.23 (0.16,2.17)	0.72	2.09(1.39,2.84)	1.0	-0.04(-1.03,1.18)	0.26
	GA (*n* = 56;50.9)	1.83(0.95,2.38)	1.0	1.22(0.43,1.92)	0.93	2.84(1.57,3.53)	1.0	1.59 (0.2,2.28)	0.72	1.90(1.45,2.73)	1.0	-0.21(-0.65,0.92)	0.24
	AA (*n* = 23;20.9)	1.70(0.95,2.42)		1.06(0.60,1.92)		2.47(1.61,3.49)		1.23 (0.42,1.59)		1.81(1.27,2.68)		-0.41(-1.24,0.26)	

^1^% *n*-3 HUFA levels are expressed as the median (inter-quartile range) value

^2^for the comparison with AA homozygote levels by the Kruskal-Wallis test

^3^delta values are 6-month or 12-month % *n*-3 HUFA subtracted by the respective baseline value. Not all participants that received EPA and who provided a baseline blood sample (*n* = 147) gave a blood sample at 6 or 12 months so Δn-3 HUFA values are based on *n* = 35 (GG), *n* = 58 (GA) and *n* = 23 (AA) at 6 months and *n* = 31 (GG), *n* = 56 (GA) and *n* = 23 (AA) at 12 months

^4^data are expressed as the median (IQR) of individual participant Δ*n*-3HUFA values

^5^for the comparison of 6- or 12-month values for the respective change from baseline with AA homozygote values by the pairwise Wilcox test with Benjamini-Hochberg adjustment

^6^only seAFOod trial participants randomised to EPA alone

At baseline, prior to trial intervention, minor (A) homozygotes displayed higher levels of *n*-3DPA and DHA in RBCs than major (G) allele carriers, which reached statistical significance for *n*-3DPA content (Table 1). Corresponding median (IQR) *n*-3DPA/EPA ratios were 2.55 (1.91, 3.13) in AA homozygotes, 2.34 (1.75, 3.03) in GA heterozygotes, and 2.34 (1.79, 2.93) in GG homozygotes, which did not differ significantly (*P* = 0.14). These results are consistent with increased ELOVL2-dependent conversion of EPA to longer-chain *n*-3DPA in AA homozygotes compared with G allele carriers (Table 1).

For analysis of the relationship between *ELOVL2* genotype and RBC *n*-3 HUFA levels after the start of the seAFOod trial intervention, we restricted analysis to participants who received EPA only (Table 1). As expected, the rs953413 genotype frequency of participants, who received EPA treatment only and provided *n*-3 HUFA data at 6 and 12 months after starting the trial intervention, was similar to the rs953413 genotype frequency of the larger cohort of 603 trial participants who provided baseline data (Table 1). We analysed the absolute RBC levels of *n*-3 HUFAs at each time point, as well as the change in % n-3 HUFA content of RBCs between baseline and after either 6 or 12 months of treatment (Δ*n*-3 HUFA).

**Fig. 1:**
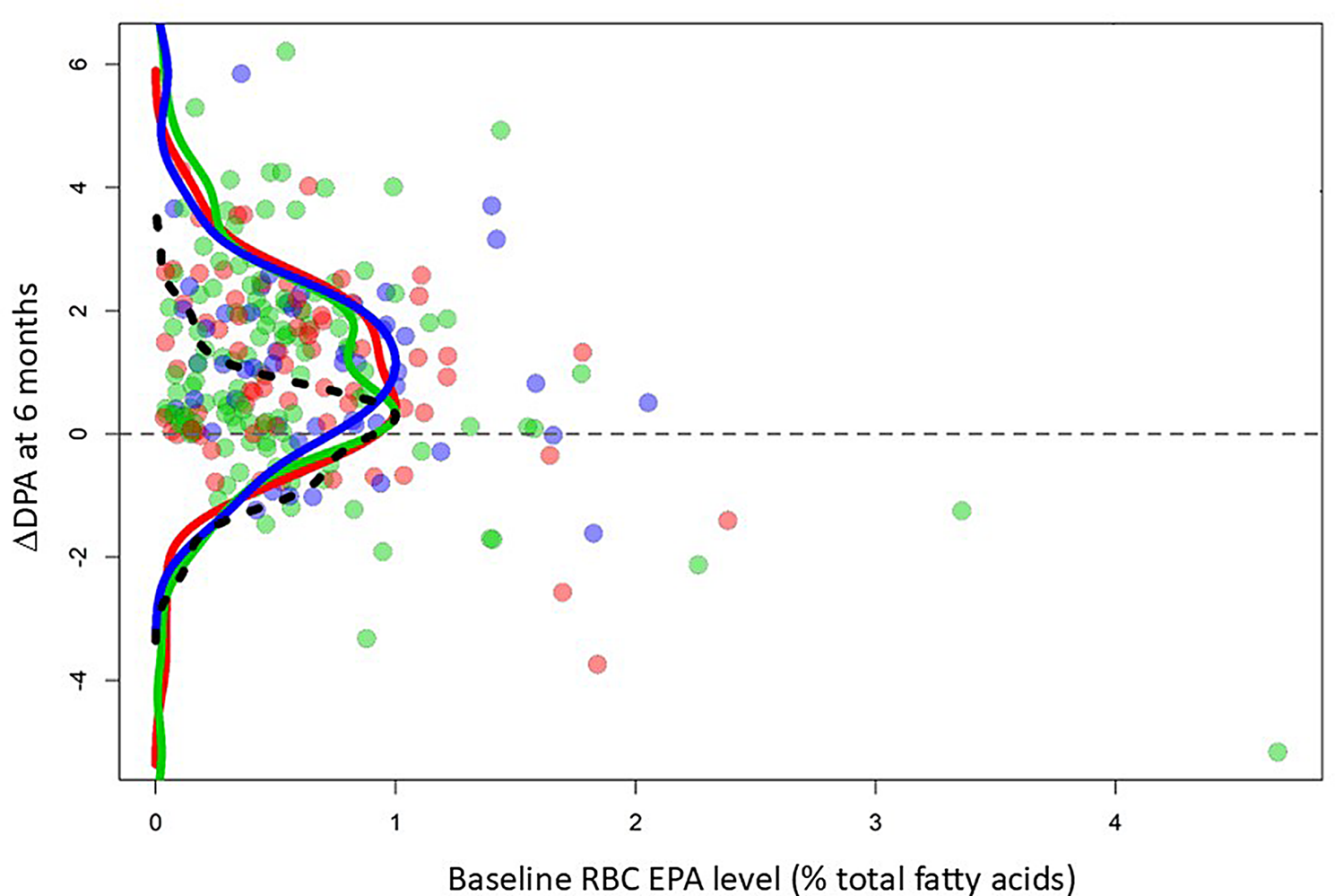
The change in RBC *n*-3DPA level in EPA users during seAFOod trial participation according to *ELOVL2* SNP rs953413 genotype. The difference in RBC DPA level after treatment for 6 months (V4) compared with baseline (V1) is plotted at individual participant-level according to the baseline RBC EPA value, consistent with previous seAFOod trial analysis [2]. Density curves represent the distribution of data points for participants that were major (G) allele homozygotes (red), heterozygotes (green) or minor (A) homozygotes (blue). The black dashed density curve represents data from placebo EPA users for comparison with the data from individuals randomised to active EPA with different *ELOVL2* genotypes. Density curves show a more pronounced upward shift in ΔDPA values in AA homozygotes (blue) compared with GG homozygotes (red) or heterozygotes (green), which both have more overlap with the density curve for placebo-users (black). Note that the green density curve for GA heterozygotes is bimodal, unlike those for homozygous individuals

We already reported that participants who received EPA in the seAFOod trial demonstrated an increase in RBC levels of EPA and *n*-3DPA, but not DHA, compared with those allocated to placebo capsules [2]. Here, we analysed *n*-3 HUFA levels only in EPA users stratified for rs953413 genotype. Figure 1 shows individual Δ*n*-3DPA values at 6 months plotted against the respective baseline RBC EPA value, consistent with our previous report of RBC Δ*n*-3HUFA values for the whole seAFOod trial population during EPA treatment [2]. After 6 months of EPA treatment, participants who were AA homozygotes displayed an upward shift in the distribution of Δ*n*-3DPA values (blue density curve) compared with placebo users (black density curve). The increase in *n*-3DPA values in AA homozygotes (blue density curve) was higher than the shift of corresponding density curves for GA heterozygotes (green) or GG homozygotes (red) that both overlapped more with the summary curve peak (black) for individuals who received placebo capsules only (Fig. 1). However, the differences in Δ*n*-3DPA values according to rs953413 genotype did not reach statistical significance (Table 1). There was also no significant difference between the change in RBC EPA or DHA levels after 12 months of EPA treatment according to rs953413 genotype (Table 1).

## Discussion

In this secondary analysis of the seAFOod polyp prevention trial, we found that minor (A allele) homozygotes for the *ELOVL2* SNP rs953413 have a higher baseline RBC *n*-3DPA level than G allele carriers, consistent with increased elongation of EPA by ELOVL2. However, rs953413 genotype did not significantly influence ΔEPA, Δ*n*-3DPA, or ΔDHA levels during 6 or 12 months of high-dose EPA supplementation. We do not provide evidence that genetic variation in *ELOVL2* provides a major contribution to the inter-individual variability in the circulating EPA level associated with EPA supplementation. The influence of SNP rs953413 was evident for *n*-3DPA levels, but not for EPA or DHA levels. This observation is consistent with multi-step enzymatic synthesis of EPA and DHA from precursor C18:3*n*-3 alpha-linolenic acid, which involves two fatty acid desaturase genes and ELOVL5, in addition to ELOVL2 [9].

Our results align partly with prior studies linking *ELOVL2* polymorphisms to differences in long-chain *n*-3 HUFA levels. A scoping review of the relationship between *ELOVL* polymorphisms and blood levels of EPA and DHA in 2024 identified six studies that have previously investigated the relationship between *ELOVL2* polymorphisms (four studies that included rs953413 genotyping) and blood *n*-3 HUFA levels [3]. There was an association between the presence of at least one minor allele of the *ELOVL2* polymorphism and lower plasma EPA or DHA level in three (50%) of the studies (two included rs953413) with the remainder revealing no association with plasma *n*-3 HUFA levels [3]. It should be noted that we reported RBC levels of n-3 HUFAs, whereas the majority of the previous studies measured plasma levels of n-3 HUFAs. Variability in genetic association studies related to different *n*-3 HUFA pools is highlighted by comparison of *n*-3 HUFA data from the InCHIANTI (plasma) and GOLDN (RBC) cohorts, which reported lower and no difference in EPA levels according to rs953413 genotype, respectively [10].

The absence of an association between rs953413 genotype and the change in RBC EPA level during EPA supplementation in our study indicates that other host factors are more important determinants of tissue EPA levels during *n*-3 HUFA supplementation. These include age, sex, background dietary PUFA intake, and baseline circulating *n*-3 PUFA level [2, 11, 12]. The relationship between genetic variants in other PUFA biosynthetic genes involved in conversion of EPA to DHA (*ELOVL5* and *FADS2*) and response to EPA supplementation has yet to be addressed [9].

Strengths of this study include the well-characterised cohort and standardised EPA dosing in the context of a randomised trial, as well as measurement of RBC HUFA levels. Limitations of this secondary trial analysis include the modest size of the study, thereby limiting the number of AA homozygotes, restriction of genetic variants to five *ELOVL2* SNPs, and reduced generalisability to racially diverse populations given that the seAFOod trial cohort was White European [7].

In conclusion, *ELOVL2* SNP rs953413 minor homozygosity is associated with higher baseline RBC *n*-3DPA levels but does not significantly modify the change in circulating EPA level in response to high-dose EPA supplementation for 12 months.

## Data Availability

The genotype and *n*-3 HUFA datasets described in this report contain identifiable data, which are stored in a secure trusted research environment at the University of Leeds. Data will be anonymised and exported upon request to the Principal Investigator (Professor Hull) and the Study Sponsor (University of Leeds).

## Abbreviations

BMI: Body mass index
DHA: Docosahexaenoic acid
DPA: Docosapentaenoic acid
ELOVL: Elongation of very long-chain fatty acids
EPA: Eicosapentaenoic acid
FFA: Free fatty acid
FFQ: Food frequency questionnaire
HWE: Hardy-Weinberg equilibrium
HUFA: Highly unsaturated fatty acid
IMP: Investigational Medicinal Product
IQR: Interquartile range
LC-MS/MS: Liquid chromatography-tandem mass spectrometry
MPP: Mean polyp number per participant
PDR: Polyp detection rate
RBC: Red blood cell
SACN: Scientific Advisory Committee on Nutrition
SAT: Saturated fatty acid
SD: Standard deviation
SNP: Single nucleotide polymorphism
TG: Triglyceride
V: Visit
